# Systematic Characterizations of Text Similarity in Full Text Biomedical Publications

**DOI:** 10.1371/journal.pone.0012704

**Published:** 2010-09-15

**Authors:** Zhaohui Sun, Mounir Errami, Tara Long, Chris Renard, Nishant Choradia, Harold Garner

**Affiliations:** 1 Virginia Bioinformatics Institute, Blacksburg, Virginia, United States of America; 2 Department of Math and Natural Sciences, Collin College, Plano, Texas, United States of America; Universidad Peruana Cayetano Heredia, Peru

## Abstract

**Background:**

Computational methods have been used to find duplicate biomedical publications in MEDLINE. Full text articles are becoming increasingly available, yet the similarities among them have not been systematically studied. Here, we quantitatively investigated the full text similarity of biomedical publications in PubMed Central.

**Methodology/Principal Findings:**

72,011 full text articles from PubMed Central (PMC) were parsed to generate three different datasets: full texts, sections, and paragraphs. Text similarity comparisons were performed on these datasets using the text similarity algorithm eTBLAST. We measured the frequency of similar text pairs and compared it among different datasets. We found that high abstract similarity can be used to predict high full text similarity with a specificity of 20.1% (95% CI [17.3%, 23.1%]) and sensitivity of 99.999%. Abstract similarity and full text similarity have a moderate correlation (Pearson correlation coefficient: −0.423) when the similarity ratio is above 0.4. Among pairs of articles in PMC, method sections are found to be the most repetitive (frequency of similar pairs, methods: 0.029, introduction: 0.0076, results: 0.0043). In contrast, among a set of manually verified duplicate articles, results are the most repetitive sections (frequency of similar pairs, results: 0.94, methods: 0.89, introduction: 0.82). Repetition of introduction and methods sections is more likely to be committed by the same authors (odds of a highly similar pair having at least one shared author, introduction: 2.31, methods: 1.83, results: 1.03). There is also significantly more similarity in pairs of review articles than in pairs containing one review and one nonreview paper (frequency of similar pairs: 0.0167 and 0.0023, respectively).

**Conclusion/Significance:**

While quantifying abstract similarity is an effective approach for finding duplicate citations, a comprehensive full text analysis is necessary to uncover all potential duplicate citations in the scientific literature and is helpful when establishing ethical guidelines for scientific publications.

## Introduction

Computational methods have proven effective in the identification of highly similar and potentially unethical scientific articles. In our previous study, the text similarity-based information retrieval search engine eTBLAST [1] was tuned with the MEDLINE abstract dataset [2] to create Déjà vu, a publicly available database of over 70,000 highly similar biomedical citations [3]. The abstract of each MEDLINE citation was compared to its top related article in MEDLINE (a feature available from MEDLINE) using eTBLAST. The citation pairs with similarity ratios exceeding the calibrated threshold were deposited into the Déjà vu database [3]. Subsequently, the computationally discovered similar citation pairs were manually examined by several curators to verify, classify, and characterize them [3]. The ongoing analysis of entries in Déjà vu has uncovered several unethical publication practices ranging from co-submission to plagiarism to data falsification [4], [5]. However, our current computational method is not without limitations. Because it utilizes only abstracts to find similar citations, it inevitably omits potential duplicate full text articles whose abstracts may not appear similar enough to warrant further investigation.

Full text articles have become increasingly available via PubMed Central (PMC), NCBI's free digital archive of biomedical and life sciences journal literature. As of October 2009, there are 785 journals indexed in PMC whose archives of full text articles are freely available on the web (http://www.ncbi.nlm.nih.gov/pmc/index.html). The electronic availability of such manuscripts has aided in the identification of duplicate citations by allowing for more transparency and thus more precise characterizations of the similarities amongst these articles.

Our previous publications regarding scientific integrity [4], [5] through the duplicate findings in Déjà vu have stimulated a broad range of discussions on scientific ethics [6], [7]. Although individual thoughts on this topic vary, a general consensus can be drawn that scientific publication standards are simply not well established enough to account for all types of dubious behaviors [7]. The systematic full text similarity analysis performed in this study will help quantify the current trends and behaviors of duplicate publication and ultimately aid the scientific community in forming more rigid standards concerning unethical practices and their respective consequences.

In this study, we established a new, more precise method of finding duplicate citations by using text similarity algorithm eTBLAST to analyze PMC's database of full text articles. Using this method, we have systematically characterized the text similarity in several data sets generated from the PMC full text citations. All the data sets generated from the full text PMC citations in this study are available on http://eTBLAST.org for text similarity comparisons.

## Materials and Methods

### Text similarity comparison tools

The eTBLAST tool (http://eTBLAST.org) was originally designed as a search engine to retrieve relevant biomedical literature [1], and has been successfully used to search MEDLINE abstracts with whole paragraph queries.

The eTBLAST-based text similarity comparison methods described in previous studies [2] were applied to pairs of full text articles from PMC. Briefly, similarity scores were calculated by comparing one set of text (query) against another set of text (subject) using eTBLAST. An identity score was computed by comparing the subject text against itself. The similarity ratio for the pair was simply the similarity score divided by the identity score. We previously found that, when classifying pairs of text as potential duplicates, a similarity ratio cut-off of 0.5 achieved a good specificity and sensitivity [2]. The same cut-off value was applied when identifying duplicate text pairs from PMC. An eTBLAST API (http://eTBLAST.org/interface) was created to facilitate a programmatic interface with the core eTBLAST text comparison engine.

### Text data sets

Full text datasets were downloaded from PubMed Central (http://www.ncbi.nlm.nih.gov/pmc/about/ftp.html). A Perl program was written to parse the XML files, extract text from each citation, divide the text into separate sections (Introduction, Methods, and Results/Discussion) as well as separate paragraphs, and use these to create different text datasets. The eTBLAST algorithm is optimized to identify similarities among text whose size is roughly equivalent to that of a Medline abstract [1], [2]. Because PubMed Central's full text articles are usually much longer than their abstracts, dividing the full text articles into smaller parts (i.e., sections and paragraphs) allows eTBLAST to compare them more efficiently and effectively.

A total of 107,205 citations were retrieved from PubMed Central, 72,011 of which contained full text articles. We created 3 different granularities of text data sets: full text, sections, and paragraphs. Classifying the sections by matching key words in the section titles, we retrieved 61,149 introduction sections, 50,363 method sections, and 135,063 results/discussion sections (some articles have more than one results/discussion section). Some articles did not have either the typical introduction-methods-results section structure or section header key words, and therefore were not included in the section data sets. We further divided all of the sections into 2,719,231 paragraphs and categorized them as either introduction paragraphs, methods paragraphs, or results/discussion paragraphs, using the same classification strategy that was used for sections. The abstracts of all the citations were retrieved as well.

### Indices and similarity comparisons

We built several PubMed Central text indices from the data sets described above. Using these indices, we performed text similarity comparisons for every set of text among the three levels of granularity. We also compared the abstracts of the citations. Several Perl scripts were developed to perform the batch eTBLAST searches for the text in all of the data sets. When the text comparisons for a data set were performed, each individual text in the set was compared against all other texts in the set, and the significantly matched documents were retrieved. The average computation time for searching a 200 word text sample against the PubMed Central full text index was 2.2 seconds on a server (Dual Intel(R) Xeon CPU 2.80 GHz, 1.6 G RAM). The average computation times for searching on the section indices and the paragraph index were 0.9 seconds and 1.5 seconds, respectively.

For each of the 107,205 citations in the full text dataset, the citation was compared to all of the other citations in the full text dataset. The same text comparisons were also performed for all combinations of the three section datasets (introduction, methods, and results). Each text in the introduction, methods, and results subsets of the paragraph dataset was also compared to the entire paragraph dataset. The main measure of the similarity between a query dataset and a target dataset was the frequency of similar pairs, which is the number of similar pairs normalized by the size of the query dataset. The similar article pairs were categorized into two groups based on their authors: 1) pairs with shared authors (in which both articles share at least one common author) and 2) pairs with no shared authors (the pair of articles do not share any authors).

## Results

### Full text analysis versus abstract analysis

**Figure 1:**
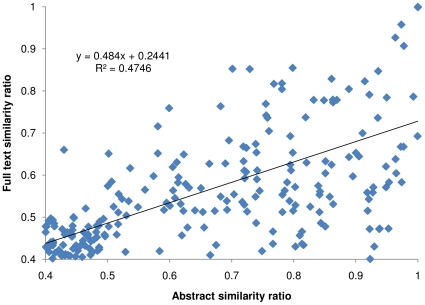
Linear regression of abstract similarity vs. full text similarity. The linear regression of full text similarity ratio versus abstract similarity ratio was performed among the citation pairs whose abstract similarity and full text similarity ratios were both higher than 0.4. The figure indicates a modest correlation between significant abstract similarity and full text similarity of citations in the similarity ratio range.

Applying a similarity ratio threshold of 0.5, we identified from the 72,011 PMC full text citations 150 citation pairs with both high abstract similarity and full text similarity, 598 pairs with high abstract similarity but no full text similarity, and 282 pairs with high full text similarity but no abstract similarity. The number of all possible pairs with neither high abstract similarity nor high full text similarity among all the PMC citations is 5.19×10^9^. Using these numbers, we evaluated the strength of association between high abstract similarity and high full text similarity in the entire PMC dataset using a log odds ratio [8] of 6.66±0.13 (confidence level 0.99). This strong association suggests that highly similar abstracts are often an indication of highly similar full text citations. Also based on these numbers, using high abstract similarity to predict high full text similarity yields a specificity of 20.1% (95% CI [17.3%, 23.1%]), sensitivity of 99.999%, and false negative rate of 1.2E-5 (95% CI [1.1E-5, 1.3E-5]). We performed the linear regression of full text similarity ratio versus abstract similarity ratio among the citation pairs whose abstract similarity and full text similarity ratios were both higher than 0.4 (Figure 1). A Pearson product moment correlation coefficient of −0.423 indicates a modest correlation between the two similarity ratios in this range.

**Figure 2:**
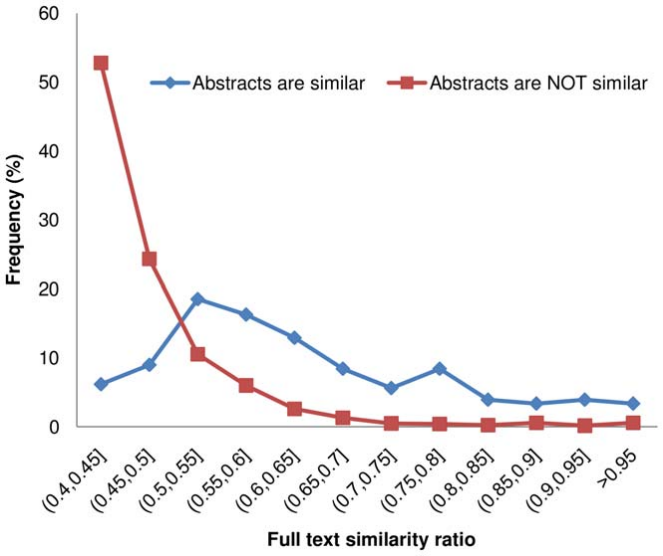
Distribution of full text similarity ratio for citation pairs with and without similar abstracts. A similarity ratio threshold of 0.5 was used to classify the abstracts as either similar or dissimilar. The figure shows that high abstract similarity is a predictor of higher full text similarity.

**Figure 3:**
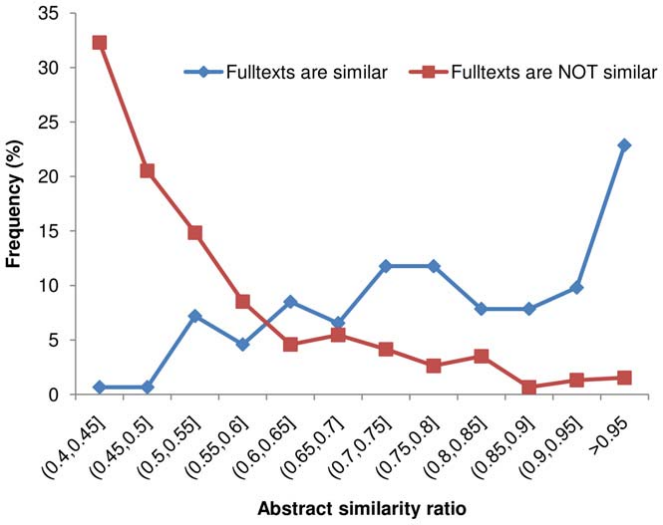
Distribution of abstract similarity ratio for citation pairs with and without full text similarity. A similarity ratio threshold of 0.5 was used to classify the full text as either similar or dissimilar. Like the trends shown in Figure 2, significant full text similarity has a correspondingly high probability of having very high abstract similarity.

We studied the full text similarity distributions of citation pairs in groups with both high and low abstract similarity (Figure 2), as well as the abstract similarity distributions of citation pairs in groups with both high and low full text similarity (Figure 3). A similarity ratio threshold of 0.5 was used to classify the citations as either similar or dissimilar. In Figure 2, for the “dissimilar abstract” group, the frequency of citation pairs drops sharply as the full text similarity increases from 0.4 to 0.55, whereas for the “similar abstract” group, the frequency of citation pairs peaks when the full text similarity is close to 0.55. In Figure 3, for the “dissimilar full text” group, the frequency of citation pairs drops dramatically as the abstract similarity ratio rises from 0.4 to 0.55, while in the “similar full text” group, the frequency of citation pairs consistently rises as the abstract similarity rises. The distribution curves of the two groups intercept in the (0.55, 0.6) range, suggesting that a value in this range may serve as an abstract similarity threshold by which one can predict high full text similarity with good specificity and sensitivity. These results are in agreement with our previous study which showed that, when using abstract similarity to find similar full text citations, an abstract similarity threshold in the (0.5, 0.6) range balances its sensitivity and specificity well [2].

### Similarity analysis among different sections of articles

We studied the association between abstract similarity and text similarity in different sections including introduction, methods and results/discussion. The conditional probabilities of having high abstract similarity given high similarity in introduction, methods, or results sections are 3.4% (sample size 87), 5.9% (sample size 846), and 9.5% (sample size 380), respectively - all multitudes higher than the probability of high abstract similarity for a random citation pair (1.44E-07). The probability of high abstract similarity given similar results/discussion sections is significantly higher than the probabilities of high abstract similarity given similar methods sections (P = 0.01) and than similar introduction sections (P = 0.03). Because the novelty of a research article is typically demonstrated more in its results/discussion sections, these findings reinforce the effectiveness of abstract text comparison in assessing the originality of scientific literature.

**Figure 4:**
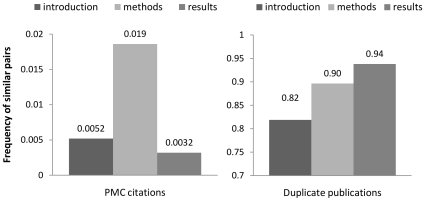
Frequency of similar pairs within different sections in PMC citations and duplicate citations. Whereas similarity in methods sections is generally more common than in other sections, similarity among results sections is the best indicator of a duplicate publication.

To better understand patterns of text repetition among different sections of articles, we computed the text similarity between paragraphs in all the introduction, methods, or results/discussion sections. We found that paragraphs in any category are most similar to other paragraphs within the same category. These results are shown in Table 1. Interestingly, the frequency of similar paragraphs within methods sections is about 3.6 times that within introduction sections and 5.8 times that within results/discussion (Table 1 & Figure 4). This demonstrates that, compared to other sections in full text biomedical literature, methods sections are the most likely to be re-used.

**Table 1:** Text similarity within different sections of articles.

	Introduction	Methods	Results
Number of documents	61149	50360	135062
Frequency of similar pairs (SA)^a^	222 (0.0036)	605 (0.012)	220 (0.0016)
Frequency of similar pairs (DA)^a^	96(0.0016)	330 (0.0066)	213 (0.0016)
Frequency of similar pairs (total)^a^	318(0.0052)	935 (0.019)	433 (0.0032)
Odds of similar pair having shared authors^b^	2.31	1.83	1.03

Duplication of methods or introduction sections is more likely committed by the same authors than duplication of results sections.

Abbreviation: SA, sharing at least one author; DA, no shared authors.

a Values are expressed as number of similar pairs (relative frequency of similar pairs).

b Values are calculated as frequency of similar pairs (SA)/frequency of similar pairs (DA).

We calculated the frequency of similar sections in a set of 193 duplicate citation pairs identified by Déjà vu, based on the manually estimated full text similarity ratio. In the dataset of real duplicates, the frequency of highly similar sections (similarity ratio >0.5) is the highest within the result sections (0.94), the second highest within methods sections, (0.89) and the lowest within introduction sections (0.82). The contrast between the PMC citation dataset and the duplicate publication dataset shows that, whereas similarity in methods sections is generally more common than in other sections, similarity among results sections is the best indicator of a true duplicate publication.

### Analysis of articles with and without shared authors

We also studied the similarities in two different types of citation pairs – pairs with at least one shared author (Same Authors - SA) and pairs with no shared authors (Different Authors - DA). In doing so, we evaluated the likelihood for a given similar text pair to have at least one shared author, using a ratio calculated as the number of SA pairs over the number of DA pairs. That is, we calculated the odds of similar articles in a pair having at least one shared author (Table 1). Although any given article in the PMC dataset can be compared to 72,010 other articles, the average number of article pairs with at least one shared author is 5.87. For a random article pair in PMC, the odds of both articles sharing at least one author is smaller than 6/72010 = 8.33E-05. The likelihood of a similar text citation pair to share at least one author (odds  = 458/276 = 1.66) is significantly greater than that of a random pair. Furthermore, as shown in Table 1, for a citation pair with similarities in the introduction or methods sections, the odds of having at least one shared author (introduction: 2.31, methods:1.83) is 154% and 101% higher, respectively, than that for a citation pair with results similarity (1.03). In other words, duplications of methods or introduction sections are more likely committed by the same authors than are duplications of results sections.

### Analysis of review articles

There are 5,414 review articles in our PMC datasets. We surveyed the similarity among pairs of reviews and pairs containing one review and one non-review (original research) article. The frequency of similar pairs is 0.0167 for review-to-review comparisons and 0.0023 for review-to-non-review comparisons. If we inspect the similarity between review articles and original research articles, reviews are most similar to the results sections of other original articles (944 similar pairs with respect to 512,739 total result paragraphs, ratio≈0.0018), versus a ratio of 0.001 (281/296,757) for introduction sections and 0.0005 (315/582,267) for methods sections.

We also analyzed the likelihood of any given pair of similar reviews to contain at least one shared author. Similar review pairs were identified using paragraph level text comparisons. We found that the odds of having at least one shared author is very low (0.089) in the set of similar pairs containing at least one review, while the odds of having at least one shared author among similar pairs in the entire PMC set is significantly higher at 0.78. In other words, similar citation pairs in which at least one of the articles is a review are much more likely to have been produced by different authors. Interestingly, the odds of a similar pair with one review and one original research article having at least one shared author (0.05) is much lower than that of a similar pair of review articles (0.58). Of the 262 similar pairs of review articles with at least one shared author we observed, 177 (67.6%) were published in the same journals and 142 (54.2%) were published within the same year.

## Discussion

Abstract similarity analysis of MEDLINE citations was previously used to detect potential duplicate publications [2], [3]. However, abstract similarity alone is not necessarily predictive of full text similarity or sections therein, and thus full text analysis is needed to give a thorough and comprehensive picture of the complete text similarity. To demonstrate this, we generated a list of manually discovered article pairs in Déjà vu [3] with high full text similarity but very low abstract similarity (Table S1). This study has shown abstract similarity to be a good predictor of full text similarity. While abstract comparison remains a useful tool for finding similar citations, with the ever-expanding availability of full text scientific literature on the web, full text comparison will become increasingly important in the identification of duplicate publications. eTBLAST now allows users to search for text-similar articles by comparing MEDLINE abstracts and PMC full text articles. Very high text similarity (e.g., over 85%) identified by eTBLAST could suggest a case of plagiarism if the pair of articles do not share any authors, or co-submission if the pair of articles share at least one author. After the highly similar pairs are identified by eTBLAST, manual examination should be done to verify their amounts of full text similarity and determine whether or not the duplicates are legitimate cases (e.g., update, re-publication by journals) before reporting the cases [5].

Studies have repeatedly shown duplicate publication by the same authors to be a rising problem [2], [4], [7], [9]. In order to understand the behavior, it must first be measured in a systematic and quantitative way. Anecdotal evidence on a case-by-case basis indicates that certain sections of papers (e.g., Introduction and Methods) are copied more frequently than others [7], but it is not clear to what extent or in what patterns this follows. Our comprehensive survey of the text similarity among different sections of biomedical articles, both with and without shared authors, helps quantify and ultimately aid in understanding the nature of duplication in our scientific literature. Our findings regarding the abundance and repetitive nature of review articles once again raises a question that journal editors and policy makers have been asking for years – are there too many reviews? The scientific community should make an effort to define clear guidelines on publishing scientific literature in order to prevent future unethical publications. Journals can play important roles in defining ethical standards for scientific publications. Recently, the MEDLINE-indexed Peruvian journal *Revista Peruana de Medicina Experimental y Salud Publica* modified its Instructions for Authors after a case of duplicate publication was discovered [10]. We must also educate authors, particularly young academicians, on the appropriate practices of writing papers and publication ethics.

There were indeed limitations to this study. First, PMC's current collection of citations represents only a fraction of those in the entire MEDLINE database. Secondly, we have only manually examined a small number of the similar citation pairs identified through this method. Of the 34 highly similar pairs (full text similarity ratio >0.85) in PMC that we examined, none would be considered “unethical” by the average scientist because they were updates or multi-part publications, etc. This result is not surprising because of the small amount of pairs examined and the fact that duplicate publications tend to be published in journals with lower impact factors [5], most of which are not included in PMC at this time [11]. The average impact factor of journals containing manually verified duplicate publications with no shared authors in Déjà vu is 1.6, whereas the average impact factor of journals indexed in PMC is 2.97 [5], [11]. We should therefore expect more interesting findings from this full text analysis to emerge as a wider scope of journals are deposited into PMC.

Unethical scientific publications seem to be a problem throughout the world, and are emerging in developing countries as well as developed countries. Our previous study [4], [5] exposed several cases from developing countries such as from China, India, and Egypt. Recently, several duplicate publications and a plagiarism case in Peru and Chile were documented by Peruvian and Chilean researchers [12]–[15]. MEDLINE and PMC have limited collections of literature from developing countries and do not collect literature in other languages (e.g. Spanish, Chinese). Therefore, future studies should be performed using literature databases other than MEDLINE/PMC (such as SciELO) to learn more about scientific ethical issues in developing countries. It may also be worthwhile to systematically characterize the similarities among literature from other fields. For example, the physical sciences literature repository, arXiv, can be used to study the similarity and duplication in a number of physical science fields including computer science, physics, chemistry, and mathematics.

This is the first time a large-scale, comprehensive text similarity survey has been conducted on a database of full text biomedical citations. The results presented herein reinforce our previous study which showed that highly similar abstracts provide an effective means of identifying full text similar citations. Nevertheless, this study clearly demonstrates that not only are direct full text similarity comparisons needed to completely uncover all potential duplicate citations, but manual examination of the full texts is also necessary to confirm all highly similar pairs found through abstract similarity. Such undertakings will no doubt lead the scientific community to define more practical and rigid ethical guidelines for the scientific literature on which we all depend.

## Supporting Information

Table S1 Full text similar pairs in déjà vu with low abstract similarity. This table shows a list of manually inspected article pairs in déjà vu with high full text similarity but very low abstract similarity.
(0.04 MB DOC)
