# Supplementary material for: Systematic Characterizations of Text Similarity in Full Text Biomedical Publications
Source: PLoS One. 2010 Sep 15;5(9):e12704. doi: 10.1371/journal.pone.0012704 (PMC2939881; doi:10.1371/journal.pone.0012704)
Supplement: Table S1 — Full text similar pairs in déjà vu with low abstract similarity. This table shows a list of manually inspected article pairs in déjà vu with high full text similarity but very low abstract similarity. (0.04 MB DOC) [file pone.0012704.s001.doc]

**Table S1. Full text similar pairs in Déjà vu with low abstract similarity**

| Article 1 (PMID) | Article 2 (PMID) | Abstract Similarity Ratio | Estimated Full Text Similarity Ratio |
| --- | --- | --- | --- |
| 1557864 | 11396861 | <0.1 | 0.65 |
| 2470772 | 11273105 | 0.16 | 0.9 |
| 3196407 | 10617073 | 0.19 | 0.8 |
| 7575302 | 8575103 | 0.14 | 0.7 |
| 7575302 | 9122790 | <0.1 | 0.75 |
| 7658207 | 11273344 | 0.12 | 0.95 |
| 9455889 | 11952142 | <0.1 | 0.65 |
| 11000615 | 16623054 | <0.1 | 0.7 |
| 11331179 | 12512263 | <0.1 | 1 |
| 11331179 | 17021525 | <0.1 | 0.75 |
| 11602317 | 16595791 | <0.1 | 0.7 |
| 12352414 | 17951022 | 0.28 | 0.35 |
| 15448022 | 16194700 | <0.1 | 0.7 |
| 15998571 | 16619356 | <0.1 | 0.5 |
| 16025016 | 17888708 | <0.1 | 0.5 |
| 16213834 | 16728346 | 0.19 | 0.35 |
| 16816349 | 17562229 | <0.1 | 0.5 |
| 17293023 | 18178308 | <0.1 | 0.8 |
| 17650775 | 17650517 | <0.1 | 0.65 |
| 18452082 | 18718126 | <0.1 | 0.95 |

This table shows a list of manually inspected article pairs in Déjà vu with high full text similarity but very low abstract similarity.
